# The Pharmacological Options in the Treatment of Eating Disorders

**DOI:** 10.1155/2013/352865

**Published:** 2013-07-15

**Authors:** W. Milano, M. De Rosa, L. Milano, A. Riccio, B. Sanseverino, A. Capasso

**Affiliations:** ^1^Mental Health Unit, District 24, ASL Napoli 1 Centro, Molosiglio, Via Acton, 80145 Napoli, Italy; ^2^Department of Pharmacy, University of Salerno, Via Ponte Don Melillo, 84084 Fisciano, Salerno, Italy

## Abstract

The eating disorders (DCA) are complex systemic diseases with high social impact, which tend to become chronic with significant medical and psychiatric comorbidities. The literature data showed that there is good evidence to suggest the use of SSRIs, particularly at high doses of fluoxetine, in the treatment of BN reducing both the crisis of binge that the phenomena compensates and reducing the episodes of binge in patients with BED in the short term. Also, the topiramate (an AED) showed a good effectiveness in reducing the frequency and magnitude of episodes of binge with body weight reduction, both in the BN that is in the therapy of BED. To date, modest data support the use of low doses of second-generation antipsychotics in an attempt to reduce the creation of polarized weight and body shapes, the obsessive component, and anxiety in patients with AN. Data in the literature on long-term drug treatment of eating disorders are still very modest. It is essential to remember that the pharmacotherapy has, however, a remarkable efficacy in treating psychiatric disorders that occur in comorbidity with eating disorders, such as mood disorders, anxiety, insomnia, and obsessive-compulsive personality disorders and behavior.

## 1. Introduction

The eating the disorders (ED) include several pathological conditions such as anorexia nervosa (AN), bulimia nervosa (BN), the binge eating disorder (BED), and eating disorders not otherwise specified [1]. DCA are important diseases with high social impact, affecting mainly the younger members of the population with a clinical course characterized by frequent exacerbations or relapses tending to become chronic, significant sequelae medical and psychiatric comorbidities that make these diseases among the most debilitating and fatal within psychiatric [2].

The lifetime prevalence of these diseases is currently estimated at 0.6% for AN, 1% for BN, and about 3% for BED [3]. The treatment of the DCA is substantially multidimensional and includes psychotherapy, nutritional rehabilitation, and drug treatment, both to control the core symptoms of DCA and to treat frequent psychiatric comorbidities, extremely frequent in this group of pathologies [4].

Basically all the drugs introduced in the clinic were tested in an attempt to treat the clinical manifestations of eating disorders, although often with contradictory results. Among other issues, it is important to note that, with the exception of fluoxetine for the treatment of bulimia nervosa (BN), nowadays, no drug has been approved by the national and international regulatory agencies for the treatment of eating disorders. Despite the DCA have a fair impact on the population, especially in the adolescent and youth, modest findings are evidence-based literature on the pharmacological treatment of eating disorders and virtually nonexistent with regard to the treatment of children and adolescents. Also, international guidelines on pharmacological treatment of eating disorders are very small in number [5–7].

The aim of this review is focused on the use of drugs (antipsychotics, antidepressants, mood stabilizers, antiobesity, and selective norepinephrine reuptake inhibitors) in the treatment of eating disorders.

## 2. Antipsychotics

Antipsychotic drugs (AP) are used, albeit with caution, in the treatment of AN. The action of AP is mainly focused on the interaction of dopamine and serotonin systems, and often they increase appetite and weight gain in patients with major psychiatric disorders, for example, schizophrenia or bipolar disorder [8]. Paradoxically, in AN APs are not particularly useful in the weight recovery [9] but they are used to reduce other symptoms present in AN such as body image alteration, design pathologically focused on weight and food, fear of gaining weight, the obsessive-compulsive symptoms, pseudohallucinations and reduce iperarausal and agitation [10].

The first-generation antipsychotics (FGA), pimozide and sulpiride, in several randomized, placebo-controlled trials (RCTs) did not demonstrated sufficient capacity to favor weight gain [11] whereas second-generation antipsychotics (SGA) have proved more useful, in particular olanzapine, an antagonist D2/5HT2 [12]. Mondraty et al. [13], in a small sample of patients with AN, compared the olanzapine (10 mg/day) with chlorpromazine (50 mg/day) showing a net effectiveness of olanzapine in reducing the “anorexic ruminations.”

Brambilla et al. [14], in anorexia nervosa patients treated with cognitive-behavioral therapy (CBT), have compared low-dose olanzapine (2.5 mg/day in the first month and then 5 mg/day for two months) with placebo in 30 outpatients. The group treated with olanzapine reported a greater increase in weight and a significant reduction in depressive symptoms and aggression but only in patients with AN binge-purge. Bissada et al. [15] compared the olanzapine with placebo over a period of 10 weeks in a group of 34 patients with AN. The group treated with olanzapine presented a higher recovery rate of weight gain and improvement in obsessive-compulsive symptoms as measured by the Yale-Brown Obsessive-Compulsive Scale rating (YBOCS). Other SGA, such as risperidone, quetiapine, aripiprazole, and ziprasidone, have not been extensively studied in RTC trial as olanzapine in the treatment of AN [16].

These few RTC trials suggest a possible role of SGA in reducing psychiatric symptoms associated with the AN and in promoting weight recovery, but these drugs are not easily accepted among anorexics who live it as particularly intrusive and coercive respect of their personality. In addition, the possible side effects, such as extrapyramidal symptoms and QT prolongation, make them dangerous to use in frail patients, underweight, and with possible electrolyte imbalances, as are anorexic. At the moment the main international guidelines [5–7] fit the use of SGA among the secondary possibilities. There are no controlled studies on the use of antipsychotics in the treatment of BN and BED, and there are various lines of evidence that SGA may induce or exacerbate the crisis of binge eating in patients with eating disorders, as well as in psychotic patients in treatment [17].

## 3. Antidepressants

The use of antidepressant drugs (AD) in the treatment of DCA appears reasonable not only in relation to the high rates of comorbidity (greater than 50%) with mood depression in these particular patients [18] but also for the marked involvement in the genesis of eating disorders, of certain neurotransmitters such as serotonin and norepinephrine [19].

The effectiveness of AD, both of tricyclic (TCA) and of the most recent serotonin reuptake inhibitors (SSRIs), in the weight recovery in AN patients is definitely not very significant. The TCA in the few RTC literature [20] have not shown significant benefits compared to placebo. In addition, the possible side effects on the cardiovascular system of the TCA will greatly limit their use in anorexia nervosa patients. Also SSRIs, in some RCTs [21] with fluoxetine have shown little effectiveness in promoting weight regain in anorexia nervosa patients. It has been postulated that the lack of efficacy of serotonergic drugs in the acute phase of AN is modest in relation to the intake of tryptophan, the precursor of serotonin, with the power supply. In addition, Kaye [22] has suggested that there would be a poor response to AD because fasting causes an adverse effect on 5HT1a receptors and the concentration of extracellular serotonin.

In contrast, several RTC studies, systematic reviews, and meta-analyses have demonstrated the effectiveness of AD, including TCAs, SSRIs, SNRIs, and monoamine oxidase inhibitors (MAOIs) in the treatment of BN, for their ability to reduce the crisis of binge eating, purging phenomena and improve mood and anxiety [23]. Although quite effective, both MAOIs and particularly the TCA, in clinical use are not recommended for frequent and important adverse events associated with their use. Regarding SSRIs, various studies show not only the effectiveness in reducing the main symptoms of BN but also the substantial safety in their use; major concern evidence fluoxetine, citalopram, sertraline and fluvoxamine [24–26]. On average, the effective dose of SSRIs in the treatment of BN is higher than the doses used in the treatment of depression [10].

Fluoxetine has been the most studied molecule between SSRIs. A large multicenter study compared fluoxetine to placebo at a dose of 60 mg/day was superior to placebo in reducing binge crisis (67% versus 33%) and the purging phenomena (56% versus 5%) (fluoxetine BN collaborative study group, 1992). In particular, it must be remembered that fluoxetine, at the time, is the only drug approved by the FDA for the treatment of BN, at a dose of 60 mg/day.

Since the BN is a chronic disease with frequent relapses during the disease, studies over short periods have limited clinical value. Unfortunately, most of the evidence of efficacy is focused on trials lasting several months, even for the low compliance to drugs of this type of patients [27]. The RTC study with the greater duration of observation, 58 weeks, has demonstrated the efficacy of fluoxetine, compared to placebo, not only in reducing the episodes of binge and purging, the obsessive-compulsive symptoms but also in reducing the frequency of relapses, even though the attrition rate was high, 83% of patients treated with fluoxetine compared with 92% with placebo [28].

The AD also proved to be useful in the treatment of binge eating disorder (BED) both decreasing *n* binge seizure frequency and improving symptoms of depression and anxiety often present in BED. Two meta-analyses [29, 30], which together involved more than 600 patients with BED, showed that treatment with SSRIs favored, compared with placebo, a significant reduction in binge crisis. The same meta-analyses, however, have reported a modest effect of SSRIs used, compared to placebo in reducing the body weight of the patients. Even duloxetine, an AD with action reuptake inhibitor of both serotonin and noradrenaline (SNRI), has shown in a recent randomized controlled trial of 40 patients with BED, the ability to reduce both the frequency of binge episodes crisis, body weight, and depressive symptoms [31].

Another drug acting similar to AD, sibutramine, an antiobesity and antireuptake of serotonin and norepinephrine, has shown in two RCTs [32, 33] a significant effect in reducing both the binge crisis and the body weight compared to placebo. In recent years, due to the possible occurrence of adverse cardiovascular nature, sibutramine has been withdrawn from the European and the United States markets. Among the various AD, bupropion seems contraindicated in the treatment of BN and BED for the possible risk of inducing seizures [34].

## 4. Mood Stabilizers

In recent years, numerous studies have suggested the use of different antiepileptic drugs (AEDs) in the management of some forms of DCA. Various reasons are the basis of these considerations. AEDs have been found useful in the treatment of various psychiatric disorders related to eating disorders, such as bipolar disorder, headache, anxiety disorders, personality disorders, and substance abuse. In addition, many drugs with antiepileptic interact with different neural systems involved in the regulation of appetite and weight nothing like the systems glutamatergic, GABAergic, serotonergic, dopaminergic, and neuropeptide Y [35, 36]. For example, valproate and pregabalin are associated with increased appetite and subsequent weight gain as topiramate, zonisamide, and fenalbato are associated with decreased appetite and weight decrease [37].

Topiramate has been shown to possess a wide spectrum of actions as antibinge eating, antipurging and to promote weight loss and therefore may be used in the treatment of both the BN that of BED. Two controlled trials have reported that the use of topiramate in the treatment of BN induced a significant reduction of the crisis of binge and purge. In the first, with an average dose of topiramate 100 mg/day, days marked by crises of binge and purge were reduced by 44.8% in patients treated with topiramate compared with 10% in those who received placebo [38, 39]. In the second topiramate, the average dose of 250 mg/day induced a significant reduction in the frequency of binge and purge, body weight, and values to the SF 36 Health Survey [40].

Regarding the treatment of BED, most extensive study, a multicenter randomized controlled and with over 407 patients followed for 16 weeks, has reported that topiramate at a dose of final average of 300 mg/day induced a marked reduction in the frequency of binge episodes with significant weight loss (*P* < 0.001) and improvement of psychiatric comorbidity [41].

Topiramate, however, has some important adverse events that must be taken into account in the common clinical practice because it may induce reduction of mnemonic ability, confusion, fatigue, and impaired concentration [36].

Zonisamide, together with the CBT, has proved useful in the treatment of BED associated with obesity, in an open trial of one-year duration of 52 patients, with reduction of the manifestations binge and weight loss [42], although it presents substantially the same adverse event profile of topiramate.

## 5. Antiobesity Drugs

The orlistat, an inhibitor of gastrointestinal lipases, has been shown to be useful in reducing, albeit modestly, the symptoms of binge and facilitate weight reduction in patients with BED. At a dose of 120 mg × 3, in conjunction with a mildly hypocaloric diet, led to a weight reduction of 7.4% compared to 2.3% of placebo over 24 weeks of treatment and the number of binge crisis has not been reduced so significantly compared to placebo [43].

## 6. Drugs Acting as Selective Inhibitors of the Transport Mechanism of Presynaptic Norepinephrine

The atomoxetine has been evaluated in a controlled study lasting 10 weeks on 40 patients suffering from BED, the average final dose of 100 mg/day. The atomoxetine compared with placebo, led to a greater reduction in binge crisis, hunger, and body weight. The most common adverse events occurring with the atomoxetine were dry mouth, nausea, insomnia, nervousness, headache, and dizziness [44].

## 7. Conclusions

Pharmacotherapy of eating disorders should, in theory, not only induce a remission of core symptoms in the acute phase of the disease, but also prevent relapse over time and to be effective in treating frequent psychiatric comorbidities. At the moment, there are no effective drugs of all these clinical features. Few lines of evidence in the literature are based on randomized controlled trials. RCTs available, as we have described, showed significant limitations regarding the size of the groups of patients studied and the observation periods, rarely exceeding 12 months. Patients with eating disorders, particularly anorexia nervosa patients, often live as intrusive, aggressive, and uncontrollable with the drug treatments. Possible drugs side effects and organic comorbidities, often present in these patients, greatly reduce the long-term compliance to drug treatment. The number of dropouts in clinical studies, therefore, is considerably high.

Another critical issue is the lack of a clear dose standardization; in most of the RCTs in the literature, there is a wide variability in dosage of the same pathology. This evidence is also linked to the particular fragility of individual patients in relation to conditions of malnutrition that can lead to changes in the pharmacokinetics and pharmacodynamics of the molecules used, exposing them more to possible adverse events [8].

Basically the psychotherapies, such as CBT or family therapy, remain the treatment of choice in the treatment of eating disorders but the options offered by drug therapy have an important role in some specific eating disorders. Good lines of evidence in the literature suggest the use of SSRIs, particularly at high doses of fluoxetine in the treatment of BN and in reducing the episodes of binge in patients with BED in the short term while there are still few data on the efficacy for periods exceeding 54 weeks. Various studies have shown that treatment with SSRIs increases the effectiveness of CBT on the core symptoms of BN and BED.

Even for the topiramate (an AED), there are discrete evidence of effectiveness in reducing the frequency and magnitude of binge episodes with body weight reduction, both in the BN and in the BED therapy. To date, few data support the use of low doses of second-generation antipsychotics in an attempt to reduce the design focused on weight and shapes, the obsessive component, and anxiety in anorexic patients. Finally, we must remember that only fluoxetine, at the moment, is authorized by regulatory authorities for the treatment of BN and no drugs for the treatment of AN or BED.

The drug therapy, however, has a remarkable efficacy in treating psychiatric disorders that occur in comorbidity with eating disorders, such as mood disorders, anxiety, insomnia, and obsessive-compulsive, personality disorders and behavior.

In conclusion, we can state that significant progress has been made in recent decades in research on drug therapy in the treatment of eating disorders. There are, however, necessary methodological improvements that include assessments of outcomes not only on the weight, eating behavior, and the phenomena of purging but also assessments of changes in underlying psychological and cognitive. Would be required, in addition, large-scale trials and observation periods long enough to examine combinations of treatments (psychotherapy and pharmacotherapy) in patients with severe conditions of malnutrition and comorbidities [12].

Therefore, the drugs have still a limited role, not decisive or sufficient, in the treatment of eating disorders. However, they are precious aid, and many studies have demonstrated their usefulness. In the treatment of eating disorders is necessary, as in other fields of psychiatry, associate, case by case, the best drug therapy with the most appropriate psychotherapeutic treatment, drawing from time to time, the intervention strategy as a function of the type of pathology (indicators diagnostic) and individual characteristics of the patient (clinical indicators) [45, 46].

At the moment, it is clear that more and more extensive controlled studies are needed to better clarify what particular drug or combinations of drugs may be useful for specific subgroups of patients suffering from different types of eating disorders.
